# Chronic ACL Injury Drives a Fibrotic and Matrix-Degradative Shift: A Multi-Level Analysis of MMP-13 and TGF-β1

**DOI:** 10.3390/medicina62030457

**Published:** 2026-02-27

**Authors:** Yılmaz Mertsoy, Mustafa Altıntaş, Sözdar Güzel, Alpay Çetin

**Affiliations:** 1 Division of Orthopedics, Gazi Yasargil Training and Research Hospital, 21090 Diyarbakır, Turkey; 2 Division of Medical Pathology, Gazi Yasargil Training and Research Hospital, 21090 Diyarbakır, Turkey

**Keywords:** anterior cruciate ligament, ACL remnant, MMP-13, TGF-β1, fibrosis, extracellular matrix, QuPath, immunohistochemistry, clinical outcomes, bioinformatics

## Abstract

*Background and Objectives*: The biological state of anterior cruciate ligament (ACL) remnant tissue may influence postoperative healing, yet the molecular changes associated with injury chronicity remain poorly defined. This study evaluated MMP-13 and TGF-β1 expression in human ACL remnants to characterize their regenerative or fibrotic potential. *Materials and Methods*: ACL remnants from acute (<3 months) and chronic (>6 months) injuries were analyzed using histology, immunohistochemistry, and QuPath-based digital quantification. Clinical outcomes were correlated with marker expression. Protein–protein interaction and KEGG enrichment analyses were performed to identify extracellular matrix (ECM)-related pathways associated with MMP-13 and TGF-β1. *Results*: Chronic ACL remnants exhibited disorganized ECM structure with significantly higher MMP-13 and TGF-β1 expression across all digital metrics, including DAB-positive area, cell density, optical density, and H-score (*p* < 0.01). Higher expression of both markers correlated with lower IKDC and Lysholm scores and greater residual pivot-shift positivity. Bioinformatic analysis identified 39 shared proteins enriched in ECM-receptor interaction, TGF-β signaling, and fibrosis-related pathways, aligning with the degenerative phenotype observed in chronic tissue. *Conclusions*: ACL remnant biology evolves from a reparative profile in acute injuries to a fibrotic, matrix-degradative state in chronic injuries. MMP-13 and TGF-β1 serve as indicators of remnant quality and may help guide timing of surgery and future biologic strategies aimed at improving ACL reconstruction outcomes.

## 1. Introduction

Anterior cruciate ligament (ACL) rupture is one of the most common knee injuries among young and active individuals, accounting for nearly half of all ligamentous knee traumas [1]. Despite advances in surgical reconstruction techniques, the biological environment at the time of surgery remains a critical determinant of postoperative healing, graft integration, and long-term joint stability [2]. The ACL possesses limited intrinsic healing capacity due to its intra-articular location, poor vascularity, and rapid loss of fibrin scaffold formation, all of which contribute to progressive degeneration of the torn remnant over time [3]. The incidence of anterior cruciate ligament injury is influenced by multiple intrinsic and extrinsic risk factors, including sex-related differences, level and type of sports participation, neuromuscular control, and anatomical predispositions such as tibial slope and intercondylar notch width [4]. Non-contact mechanisms predominate and typically involve sudden deceleration, pivoting, knee valgus collapse, and improper trunk control during landing or cutting maneuvers [5,6].

In recent years, the ACL remnant has attracted growing attention because of its potential role in enhancing graft revascularization, maintaining proprioception, and providing biological support during reconstruction [7]. However, accumulating evidence suggests that the quality of the remnant is highly time-dependent and that its molecular profile may shift from a regenerative to a degenerative state as injury chronicity increases [8]. Transcriptomic analyses have shown that early ACL tears demonstrate activation of genes related to tissue repair, angiogenesis, and extracellular matrix (ECM) synthesis, whereas chronic tears show enrichment of inflammatory, catabolic, and fibrotic pathways [9]. These findings indicate that the biological value of the remnant is not uniform and may decline with delayed surgical intervention.

Two molecular pathways are particularly relevant to this shift: matrix metalloproteinase-13 (MMP-13) and transforming growth factor-beta 1 (TGF-β1). MMP-13 is a potent collagenase responsible for degrading fibrillar collagens, especially type I and type II, and plays a central role in ligament degeneration, osteoarthritis progression, and chronic tendon remodeling [10,11]). Its overexpression has been implicated in structural weakening of ligament tissue and impaired biomechanical function. On the other hand, TGF-β1 is a multifunctional cytokine that regulates ECM deposition, fibroblast activation, angiogenesis, and scar formation [12]. While TGF-β1 promotes early tissue repair, sustained activation leads to fibrosis, abnormal collagen accumulation, and loss of functional tissue architecture [13]. Excessive TGF-β1 signaling has been documented in a variety of fibrotic musculoskeletal disorders and may similarly contribute to maladaptive remodeling in chronically torn ACL tissue [14].

Emerging data suggest that the interplay between MMP-13 and TGF-β1 may create a persistent cycle of ECM degradation and fibrosis, driving chronic transformation of the ACL remnant [15]. Fibroblasts exposed to prolonged TGF-β1 stimulation can simultaneously upregulate MMPs, promoting disorganized collagen turnover and scar-like remodeling [16]. Such a dual pro-fibrotic and matrix-degradative microenvironment may undermine the structural and biological integrity of the remnant and potentially influence surgical outcomes. Clinically, poor-quality remnants have been associated with inferior postoperative stability, reduced proprioception, and lower patient-reported functional scores [17,18]. Given the increased adoption of remnant-preserving ACL reconstruction and the ongoing debate surrounding the optimal management of the remnant, a clearer understanding of its molecular evolution is needed. Although previous studies have examined gene expression patterns across varying injury intervals, few have integrated histological, immunohistochemical, digital quantitative analysis, clinical correlations, and bioinformatic pathway exploration to comprehensively characterize the biological state of the ACL remnant.

The aim of this study was therefore to investigate the histopathological and molecular characteristics of human ACL remnants in acute and chronic injuries, with a particular focus on MMP-13 and TGF-β1 expression. By combining immunohistochemistry, QuPath-based digital quantification, clinical outcome analysis, and ECM-related bioinformatic profiling, we sought to elucidate the biological trajectory of the ACL remnant and its potential implications for surgical timing, graft integration, and remnant-preserving strategies.

## 2. Materials and Methods

### 2.1. Study Design

This study was conducted as a retrospective analysis of ACL remnant tissues collected during routine arthroscopic ACL reconstruction procedures performed between January 2023 and December 2024. Ethical approval for the retrospective use of archived human tissue samples and associated clinical data was obtained from the Clinical Research Ethics Committee of Gazi Yaşargil Training and Research Hospital (Approval No: 2025/725). All procedures were conducted in accordance with the ethical standards of the institutional research committee and with the Helsinki Declaration. Prior to sample collection, all patients were informed about the purpose of the study and the nature of the tissue use. Written informed consent was obtained from each participant. Only remnant tissues from skeletally mature patients with primary ACL rupture and no history of autoimmune disease or prior knee surgery were included. No additional intervention or procedure was performed for research purposes. Postoperative clinical outcomes, including IKDC and Lysholm scores as well as pivot-shift assessment, were evaluated at a minimum follow-up of 12 months after ACL reconstruction.

### 2.2. Tissue Samples

ACL remnant tissue was harvested from the tibial stump of the ruptured ligament during arthroscopic reconstruction using a standardized surgical technique. Approximately 5–10 mm^3^ of tissue was collected from each patient, avoiding synovial tissue contamination. All procedures were performed by orthopedic surgeon Dr. Yılmaz Mertsoy following the same operative protocol. ACL remnant tissues were collected from patients undergoing ACL reconstruction and samples were immediately fixed in 10% formalin and embedded in paraffin for histological analysis. Samples were dipped into ascending ethanol series (50%, 70%, 80%, 90%, 96% and absolute alcohol) and cleared in xylene solution. Samples were embedded in paraffin blocks and sections were cut to stain with hematoxylin eosin dye and immune staining.

### 2.3. Immunohistochemistry

Sections of 4 μm were prepared from paraffin blocks. Standard deparaffinization and rehydration protocols were followed. After antigen retrieval with citrate buffer (pH 6.0), endogenous peroxidase activity was blocked using 3% hydrogen peroxide. Sections were incubated overnight at 4 °C with primary antibodies against MMP-13 (sc-515284, 1:100 dilution) and TGF-β1 (sc-130348, 1:100 dilution) obtained from Santa Cruz Biotechnology (Dallas, TX, USA). The next day, the slides were incubated with a biotinylated secondary antibody and visualized using a DAB (3,3′-diaminobenzidine, TA-125-HDX, Thermo Fisher Scientific, Waltham, MA, USA) substrate kit. Gill III hematoxylin staining was used as a counter stain. The sections were quickly passed through the increasing ethanol series, cleared in xylene and mounted. Negative controls were performed by omitting the primary antibody, while positive controls consisted of human tissue samples known to express the target antigens, processed in parallel. All stained sections were examined and photographed using a Zeiss Axio Imager A2 light microscope (Carl Zeiss Microscopy GmbH, Jena, Germany).

### 2.4. Semi-Quantitative Scoring

Samples were grouped based on time from injury: Acute (<3 months) and Chronic (>6 months). Formalin-fixed, paraffin-embedded ACL remnant sections stained for MMP-13 and TGF-β1 were digitized at high resolution using a Zeiss Imager A2 microscope equipped with a digital camera. For each case, three non-overlapping fields representing the most cellular and morphologically preserved ligament tissue were captured at ×200 magnification and exported as TIFF images for analysis. Quantitative immunohistochemical analysis was performed using QuPath (version 0.6.0, University of Edinburgh, Edinburgh, UK). After calibration of image scale, regions of interest (ROIs) encompassing the ACL remnant were manually delineated. A color deconvolution-based “positive pixel” classifier was trained on representative slides to distinguish DAB-positive staining from hematoxylin counterstain and background. The same threshold and classifier settings were then applied uniformly to all cases. For each ROI, the following metrics were automatically extracted:

All digital analyses were performed by a pathologist experienced in musculoskeletal pathology and cross-checked by two additional blinded evaluators who were unaware of the clinical group allocation (acute vs. chronic) and patient outcomes. Interobserver agreement for ROI delineation and ordinal H-score categories was assessed in a randomly selected subset of 10 cases using Cohen’s kappa coefficient. Agreement was excellent for both MMP-13 (κ = 0.84) and TGF-β1 (κ = 0.82). Regions of interest (ROIs) were selected to represent the most morphologically preserved and cellular areas of the ACL remnant tissue, while avoiding regions with extensive artefacts, hemorrhage, or non-ligamentous tissue. ROI selection was performed independently by a pathologist experienced in musculoskeletal pathology, who was blinded to clinical data and group allocation. To minimize observer-related bias, all digital analyses were cross-checked by two additional blinded evaluators. Interobserver agreement for ROI selection and H-score categorization was assessed using Cohen’s kappa coefficient, demonstrating excellent agreement. For statistical analysis, the median of the three fields per case was used as the final value for each metric. Group comparisons between acute and chronic ACL injuries were carried out using the Mann–Whitney U test for all QuPath-derived variables, and data were expressed as median (interquartile range, IQR). A *p*-value < 0.05 was considered statistically significant.

### 2.5. Protein Network Construction and KEGG-Based Functional Enrichment of ECM-Associated Targets

To investigate the molecular interaction landscape of MMP-13 and TGF-β1 in relation to extracellular matrix (ECM) regulation, protein–protein interaction (PPI) networks were constructed using the Search Tool for the Retrieval of Interacting Genes/Proteins (STRING, version 12.0, https://string-db.org, accessed on 15 November 2025. Separate networks were generated for the Gene Ontology term “extracellular matrix organization” (GO:0030198) and for the target proteins MMP-13 and TGFB1, with a minimum interaction confidence score set to 0.4. The networks were subsequently imported into Cytoscape software (version 3.10.3), where overlapping nodes among the ECM-associated proteins and the interactors of MMP-13 and TGF-β1 were identified to determine shared regulatory proteins. To quantitatively assess the topological importance of MMP-13 and TGF-β1 within the shared-protein interactome, network centrality analysis was performed using the Network Analyzer tool in Cytoscape (version 3.10.3, National Institute of General Medical Sciences, Bethesda, MD, USA), focusing on degree centrality and betweenness centrality metrics. Following network intersection analysis, the 39 commonly interacting proteins were subjected to functional enrichment using the ShinyGO platform ((version 0.82, South Dakota State University, Brookings, SD, USA)). KEGG pathway analysis was performed using a false discovery rate (FDR) threshold of 0.05. The top 10 significantly enriched pathways were selected based on adjusted *p*-values for downstream biological interpretation.

### 2.6. Statistical Analysis

Statistical analyses were performed using SPSS (version 26.0, IBM Corp., Armonk, NY, USA). Continuous variables were assessed for normality using the Shapiro–Wilk test. Normally distributed data were compared using the independent samples *t*-test, while non-normal data, including all QuPath IHC metrics (DAB-positive area, positive cell density, mean DAB OD, H-score), were compared using the Mann–Whitney U test. Categorical variables were analyzed with the chi-square or Fisher’s exact test. Associations between MMP-13 or TGF-β1 expression and postoperative IKDC and Lysholm scores were evaluated using Spearman correlation. A *p*-value < 0.05 was considered statistically significant.

## 3. Results

### 3.1. Patient Characteristics Demonstrate Comparable Baseline Profiles Between Groups

Demographic and clinical characteristics of the patients were presented in Table 1. The acute and chronic ACL injury groups were comparable in terms of age, sex distribution, BMI, injury side, injury mechanism, and associated meniscal injuries (*p* > 0.05 for all). There were also no significant differences in preoperative functional scores, including the IKDC and Lysholm scores. However, as expected, the mean time from injury to surgery was significantly longer in the chronic group (10.4 ± 3.1 months) compared to the acute group (1.8 ± 0.9 months) (*p* < 0.001), validating the clinical grouping used in this study.

### 3.2. Acute Remnants Exhibit Preserved Structure While Chronic Remnants Show Fibrotic Degeneration

**Figure 1:**
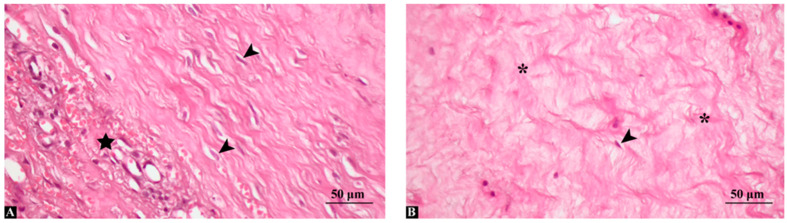
Hematoxylin and eosin staining of human anterior cruciate ligament (ACL) remnants in acute and chronic injury groups. (**A**) Acute ACL remnant tissue (<3 months post-injury) showing relatively preserved extracellular matrix (ECM) integrity, moderate fibroblast proliferation (arrowheads), and increased vascularity (star), indicating active tissue repair. (**B**) Chronic ACL remnant tissue (>6 months post-injury) displaying dense and disorganized collagen deposition (asterisks), reduced cellularity, and vascular regression. Areas of hyalinized matrix and fibrotic remodeling are evident.

Histological examination of ACL remnant tissues using hematoxylin and eosin staining revealed distinct morphological differences between the acute and chronic injury groups (Figure 1). In the acute group (<3 months post-injury), the ligament remnants exhibited relatively preserved extracellular matrix (ECM) architecture, with moderate fibroblast proliferation, increased vascularity, and mild mononuclear cell infiltration, suggesting active reparative processes. Collagen fibers appeared moderately organized, and overall tissue structure remained relatively intact (Figure 1A). In contrast, the chronic group (>6 months post-injury) showed marked fibrotic transformation characterized by dense, irregularly arranged collagen deposition, reduced cellularity, and vascular regression. The ECM appeared fragmented and disorganized, with areas of hyalinization and scar-like tissue morphology. Occasional chondroid metaplasia-like changes were also noted in some specimens (Figure 1B). These findings support a time-dependent histological transition from a reparative to a degenerative/fibrotic tissue state in the ACL remnant. Representative arthroscopic images illustrating the macroscopic appearance of ACL remnant tissue in acute and chronic injuries are provided in the Supplementary Materials, Figure S3.

### 3.3. Chronic ACL Remnants Display Stronger MMP-13 and TGF-β1 Immunoreactivity

**Figure 2:**
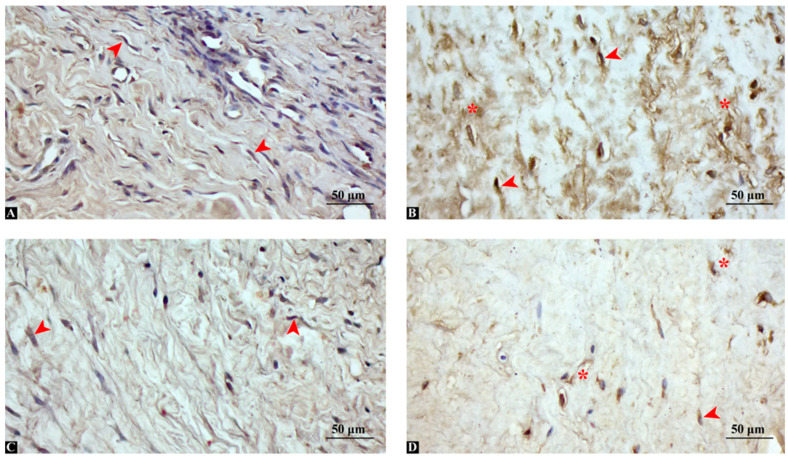
Immunohistochemical staining of MMP-13 and TGF-β1 in ACL remnant tissue from acute and chronic injury groups. (**A**) Acute ACL tissue showing weak cytoplasmic MMP-13 expression (arrowheads) in sparse fibroblasts. (**B**) Chronic ACL tissue showing strong MMP-13 immunoreactivity in fibroblast-like cells (arrowheads) and matrix-rich regions (asterisks). (**C**) Acute tissue with mild TGF-β1 staining (arrowheads) in perivascular fibroblasts. (**D**) Chronic tissue showing diffuse and intense TGF-β1 expression in fibroblast-like cells (arrowhead) and interstitial (asterisks).

Immunohistochemical analysis revealed distinct differences in the expression patterns of MMP-13 and TGF-β1 between acute and chronic ACL remnant groups (Figure 2). In the acute group (<3 months post-injury), MMP-13 expression was weak and focal, with limited cytoplasmic staining observed in a few fibroblast-like cells. Similarly, TGF-β1 expression was mild, localized primarily to perivascular regions and sparsely distributed interstitial cells (Figure 2A). Conversely, in the chronic group (>6 months post-injury), MMP-13 exhibited strong and widespread cytoplasmic positivity, particularly in fibroblast-like spindle cells and in areas of dense extracellular matrix remodeling. TGF-β1 showed intense immunoreactivity in both interstitial fibroblasts and perivascular regions, with diffuse cytoplasmic staining indicating an active fibrotic environment (Figure 2B). These findings indicate a progressive shift toward matrix degradation and fibrosis over time, with MMP-13 and TGF-β1 serving as key molecular indicators of chronic degenerative transformation in ACL remnants. MMP-13 expression was significantly higher in the chronic ACL remnant group, with strong cytoplasmic staining observed in fibroblast-like cells and matrix regions. TGF-β1 expression was also elevated in the chronic group and localized predominantly in perivascular and interstitial fibroblasts.

### 3.4. Digital Quantification Confirms Higher Matrix-Degradative and Fibrotic Marker Levels in Chronic Tissue

QuPath-based digital image analysis confirmed that both MMP-13 and TGF-β1 expression were markedly increased in chronic ACL remnants compared with acute injuries (Table 2). For MMP-13, the median DAB-positive area was more than two-fold higher in chronic tissues (36% vs. 16%, *p* < 0.01), accompanied by a substantial rise in positive cell density and staining intensity, as reflected by higher mean DAB optical density and H-scores. A similar, albeit slightly less pronounced, pattern was observed for TGF-β1, with chronic samples showing a greater proportion of DAB-positive area and significantly elevated positive cell density and H-scores (all *p* < 0.01) These quantitative findings support the semi-quantitative histopathological impression of a time-dependent shift from a relatively preserved ECM architecture in acute ACL remnants to a highly remodeled, profibrotic, and matrix-degradative phenotype in chronic lesions, characterized by strong MMP-13 and TGF-β1 activity within the ligament remnant. The distribution of QuPath-derived MMP-13 and TGF-β1 expression metrics is illustrated using box plots in the Supplementary Materials, Figures S1 and S2.

### 3.5. Elevated MMP-13 and TGF-β1 Expression Correlates with Postoperative Functional Outcomes

Expression levels of MMP-13 and TGF-β1 with postoperative outcomes were shown in Table 3. Spearman correlation analysis revealed a significant negative association between MMP-13 expression in ACL remnants and postoperative IKDC scores (r = −0.46, *p* = 0.009) as well as Lysholm scores (r = −0.41, *p* = 0.018). Similarly, higher TGF-β1 expression correlated with lower IKDC (r = −0.42, *p* = 0.015) and Lysholm scores (r = −0.38, *p* = 0.026), indicating that increased matrix-degrading and profibrotic activity was associated with inferior functional outcomes.

Patients were stratified according to median marker expression in Table 4. Patients in the high MMP-13 group exhibited significantly lower median IKDC and Lysholm scores compared with the low MMP-13 group (*p* = 0.012 and *p* = 0.018, respectively). A similar pattern was observed for TGF-β1, with high-expression patients showing poorer clinical scores than those with low expression (both *p* < 0.05). Moreover, the proportion of patients with a positive pivot-shift test at final follow-up was higher in the high-expression groups for both markers (*p* < 0.05), supporting the notion that chronic matrix remodeling and fibrosis in ACL remnants are linked to residual joint laxity and suboptimal functional recovery.

### 3.6. Bioinformatic Analysis Reveals ECM-Related Fibrotic Pathways Shared by MMP-13 and TGF-β1

**Figure 3:**
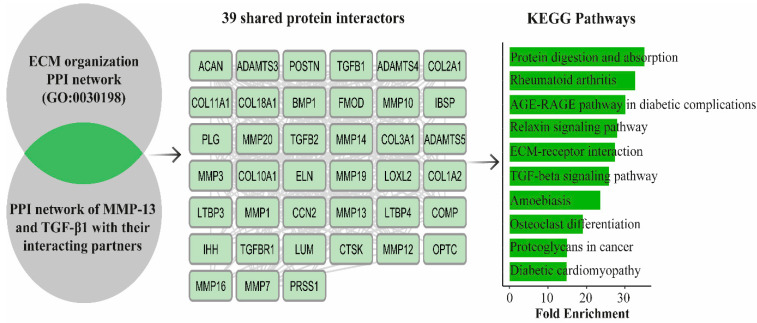
KEGG pathway enrichment analysis of the 39 shared proteins between the ECM organization network and the interactors of MMP-13 and TGF-β1.

The bioinformatic analyses strongly reinforced the histological, immunohistochemical, and clinical observations by demonstrating that MMP-13 and TGF-β1 converge on key molecular pathways governing extracellular matrix (ECM) remodeling and fibrotic transformation. (Figure 3) The intersected PPI network revealed that both molecules occupy central regulatory positions within a cluster of 39 shared proteins enriched for ECM organization, collagen catabolism, fibroblast activation, and tissue repair. This mo lecular profile closely mirrors the QuPath-based digital findings, in which chronic ACL remnants exhibited markedly higher DAB-positive areas, increased positive cell densities, and elevated H-scores for both markers. KEGG enrichment analysis confirmed that the predominant biological processes activated in chronic ACL tissue involve two major axes: TGF-β–driven fibrogenic signaling, including SMAD-dependent and SMAD-independent (MAPK/PI3K) pathways; and MMP-mediated matrix degradation, particularly the breakdown of fibrillar collagens mediated by MMP-13. These enriched pathways are highly consistent with the morphological features observed in chronic remnants, characterized by disorganized collagen bundles, reduced cellularity, vascular regression, and pronounced fibrosis. The simultaneous upregulation of TGF-β1 and MMP-13 suggests a coordinated shift in chronic ACL tissue toward a pathological remodeling state in which excessive collagen deposition (TGF-β1) and ongoing matrix degradation (MMP-13) coexist, ultimately leading to structural disintegration rather than regeneration. Importantly, the bioinformatic results also contextualize the clinical correlations identified in this study. Pathways such as ECM–receptor interaction, relaxin signaling, and osteoclast differentiation include multiple downstream mediators linked to tissue stiffness, impaired biomechanical properties, and persistent joint laxity. This aligns with our findings that higher MMP-13 and TGF-β1 expression levels were associated with lower postoperative IKDC and Lysholm scores and a higher incidence of residual pivot-shift positivity.

Network topology analysis revealed that MMP-13 and TGF-β1 occupy central positions within the shared-protein interactome. Specifically, MMP13 exhibited the highest degree (36 connections) and the highest betweenness centrality (0.1126), ranking first among all nodes. TGFB1 also showed high centrality values, with the second-highest degree (32 connections) and betweenness centrality (0.0678). These findings indicate that both proteins function as major interaction hubs and critical network connectors within the shared protein network (Supplementary Table S1).

## 4. Discussion

This study demonstrates that ACL remnant biology evolves markedly over time, shifting from a reparative microenvironment in acute injuries to a profibrotic and matrix-degradative state in chronic injuries. These findings align with emerging evidence that the remnant is not a static structure but undergoes progressive molecular, structural, and biomechanical changes after ligament rupture [19].

Temporal degeneration of the ACL remnant has been documented in prior histological and transcriptomic studies [20]. Brophy et al. [20] showed that chronic ACL tears exhibit increased expression of inflammatory and ECM-degrading genes, whereas earlier injuries demonstrate signatures of angiogenesis and active matrix repair. Similarly, Murray et al. [21] emphasized that the ACL’s inability to form a stable fibrin scaffold and its hypovascular intra-articular environment predispose it to chronic degeneration rather than organized healing. The structural features seen in our chronic specimens such as loss of vascularity, disorganized collagen bundles, and reduced cellularity are consistent with these observations.

The upregulation of MMP-13 in chronic ACL remnants observed in the present study parallels its known role in ligament and cartilage degradation [22]. MMP-13 is a potent collagenase responsible for cleaving type I and type II collagen and is considered a hallmark of chronic tissue remodeling [23]. Quan et al. [24] demonstrated that elevated MMP-13 disrupts fibrillar collagen alignment and compromises tissue biomechanics. Our data reinforce this concept, showing significantly increased MMP-13 expression in chronic tissue and an association between high MMP-13 levels and poorer postoperative IKDC and Lysholm scores. This suggests that excessive collagenolytic activity may diminish the structural integrity of remnants preserved during reconstruction.

TGF-β1 is a central driver of fibrosis, and its sustained expression promotes myofibroblast activity, abnormal ECM deposition, and tissue stiffening [25]. While transient TGF-β1 activation supports early healing, chronic elevation results in pathological scarring [26]. Finnson et al. [27] reported that both canonical SMAD2/3 and non-canonical MAPK/PI3K pathways contribute to TGF-β-mediated fibrosis. Our findings of diffuse and intense TGF-β1 staining in chronic ACL remnants support the notion that late-stage tissue enters a fibrotic program incompatible with functional regeneration. Similar trends have been reported in tendon and ligament healing models, where excessive TGF-β1 signaling reduces tensile strength and promotes disorganized collagen architecture [28,29].

A key contribution of the present study is the demonstration that MMP-13 and TGF-β1 are co-activated in chronic ACL tissue, suggesting a maladaptive remodeling phenotype. This duality of ongoing collagen degradation coupled with excessive fibrotic deposition creates a tissue environment that is biologically active but mechanically inferior [30]. Prior work indicates that TGF-β1 can induce MMP expression and vice versa, establishing a feedback loop that perpetuates degeneration [31]. Our KEGG pathway enrichment results, identifying ECM-receptor interaction, TGF-β signaling, and fibrosis-related pathways, support this coordinated remodeling model.

Beyond its molecular profile, the ACL remnant also has functional and biomechanical relevance [32]. Preserved remnants may contribute to proprioception, residual mechanoreceptor activity, and early graft revascularization, potentially supporting postoperative stability [33,34]. However, the biological quality of the remnant is heterogeneous and strongly influenced by injury chronicity and tissue degeneration. Chronically degenerated remnants with fibrotic and disorganized extracellular matrix architecture may lose these potential benefits and provide limited support for graft integration [35,36]. Patient-related factors such as physical activity level, joint loading, and lifestyle habits may further modulate remnant remodeling and influence both molecular expression patterns and clinical outcomes [33]. Although these factors were not systematically assessed in the present study, they should be considered when interpreting remnant biology, and future prospective studies integrating biomechanical and molecular analyses may help clarify their impact.

The clinical implications of these biological findings are substantial. Remnant-preserving ACL reconstruction has been associated with improved proprioception, vascularity, and graft maturation in some studies, but outcomes appear dependent on remnant quality [37]. Studies have shown that degenerated or fibrotic remnants may provide limited biomechanical benefit and may even hinder graft integration [21,38]. Recent clinical reports have linked poor remnant quality to persistent laxity and inferior postoperative scores [20,39]. The present study supports this notion, demonstrating that patients with high MMP-13 or TGF-β1 expression had worse IKDC and Lysholm scores and higher pivot-shift positivity. These results indicate that molecular profiling of the remnant may offer more objective guidance for surgical decision-making than arthroscopic appearance alone.

From a translational perspective, the identification of targetable pathways opens avenues for biologic modulation of the remnant. Experimental studies have explored MMP-13 inhibitors, SMAD pathway blockers, and antifibrotic agents such as relaxin for improving tendon and ligament healing [27,40,41]. Whether such approaches could enhance the biological environment of the ACL remnant, particularly in patients presenting late, needs further investigation.

Consistent with the dynamic nature of extracellular matrix (ECM) remodeling, KEGG enrichment analysis highlighted ECM–receptor interaction, TGF-β signaling, relaxin signaling, and osteoclast differentiation as key pathways [42,43]. These results support a coordinated remodeling program in which TGF-β1 acts as a central regulator of ECM production and fibrotic responses [44], while MMP-13 and related proteases contribute to ongoing matrix degradation [45]. The coexistence of structural ECM components and matrix-degrading enzymes within the shared protein network reflects an active but maladaptive synthesis–degradation balance in chronic tissue [46,47]. Collectively, these pathway- and network-level findings provide a mechanistic framework linking MMP-13/TGF-β1–centered signaling to pathological ECM remodeling. In addition to MMP-13 and TGF-β1, other molecular markers may also contribute to ligament remodeling after ACL injury. Markers such as α-smooth muscle actin (α-SMA) and tissue inhibitor of metalloproteinases-1 (TIMP-1) reflect myofibroblast activation and regulation of matrix turnover [48,49], while angiogenesis-related markers including vascular endothelial growth factor (VEGF) and CD34 are relevant to vascular remodeling and tissue repair [50,51]. Although the present study focused on MMP-13 and TGF-β1, future studies incorporating these markers may provide a more comprehensive understanding of the interplay between fibrosis, angiogenesis, and extracellular matrix remodeling during ACL remnant degeneration.

This study has several limitations. Its cross-sectional design precludes longitudinal evaluation of biological remodeling within the same patient. Mechanoreceptor density, vascular markers, ultrastructural collagen organization, and postoperative graft maturation were not assessed. Although significant correlations were observed between MMP-13 and TGF-β1 expression and clinical outcomes, multivariate regression analysis was not performed; therefore, the independent effects of these markers relative to potential confounders such as demographic factors, activity level, and associated injuries could not be fully determined. In addition, patient-related factors and region-of-interest selection in digital analysis may have influenced the findings. Finally, molecular validation using techniques such as RT-qPCR or Western blotting was beyond the scope of this study and should be addressed in future research.

In summary, the study demonstrates that chronic ACL remnants lose regenerative potential as they transition into a TGF-β–dominated fibrotic environment with high MMP-13–mediated matrix degradation. These alterations impair remnant quality and are associated with poorer postoperative function. Understanding these molecular changes can help refine indications for remnant preservation and guide the development of biologic strategies aimed at improving ACL reconstruction outcomes.

## 5. Conclusions

This study demonstrates that the biological profile of ACL remnant tissue evolves significantly with injury chronicity. Acute remnants retain features compatible with early repair, whereas chronic remnants shift toward a fibrotic and matrix-degradative state that is less favorable for healing. By integrating histological, digital, clinical, and bioinformatic analyses, our findings highlight the importance of evaluating remnant biology when planning reconstruction. Early intervention may preserve a more regenerative microenvironment, while chronic injuries may benefit from strategies targeting MMP-13 and TGF-β1 activity. These molecules emerge as meaningful indicators of tissue quality and potential therapeutic targets for improving outcomes in ACL reconstruction.

## Figures and Tables

**Table 1:** Demographic and clinical characteristics of patients undergoing ACL reconstruction, grouped by injury chronicity. No statistically significant differences were observed between the acute and chronic groups in terms of age, sex, BMI, injury side, injury mechanism, or clinical scores (IKDC and Lysholm). However, as expected, the time from injury to surgery differed significantly between groups (*p* < 0.001), confirming the temporal classification of patients.

Parameter	Acute ACL Injury (n = 60)	Chronic ACL Injury (n = 60)	*p*-Value
Age (years, mean ± SD)	26.4 ± 5.2	28.7 ± 6.3	0.274
Sex (Male/Female, n)	50/10	47/13	0.713
Body Mass Index (kg/m^2^)	24.3 ± 3.8	25.1 ± 4.2	0.593
Injury Side (Right/Left, n)	32/28	28/32	0.715
Time from Injury to Surgery			<0.001
- Mean duration (months)	1.8 ± 0.9	10.4 ± 3.1	
- Range (months)	0.5–3	7–18	
Injury Mechanism (n, %)			0.832
- Sports-related	44 (73%)	48 (80%)	
- Non-sports-related (trauma/fall)	16 (27%)	12 (20%)	
Associated Meniscal Injury (n, %)			0.452
- Present	20 (33%)	28 (47%)	
- Absent	40 (67%)	32 (53%)	
Preoperative IKDC Score (mean ± SD)	53.2 ± 9.6	49.8 ± 10.3	0.321
Preoperative Lysholm Score (mean ± SD)	64.7 ± 8.9	61.2 ± 10.1	0.287

SD: Standard deviation; n: Number of patients. Statistical test: independent *t* test.

**Table 2:** QuPath-based quantitative analysis of MMP-13 and TGF-β1 expression in ACL remnant tissues.

Marker	Group	DAB-Positive Area (%) Median (IQR)	Positive Cell Density (cells/mm^2^) Median (IQR)	Mean DAB OD Median (IQR)	H-Score Median (IQR)	*p*-Value (Acute vs. Chronic)
MMP-13	Acute ACL injury (n = 60)	16 (14–18)	120 (95–145)	0.21 (0.18–0.24)	45 (30–60)	
	Chronic ACL injury (n = 60)	36 (35–39)	260 (220–295)	0.38 (0.34–0.42)	125 (100–155)	<0.01
TGF-β1	Acute ACL injury (n = 60)	14 (12–15)	105 (85–130)	0.19 (0.16–0.22)	40 (28–55)	
	Chronic ACL injury (n = 60)	24 (22–26)	195 (165–225)	0.31 (0.28–0.35)	95 (78–120)	<0.01

**Table 3:** Correlation between MMP-13 and TGF-β1 expression in ACL remnant tissue and postoperative clinical outcomes.

Variable	MMP-13 Expression (%, Spearman r)	*p*-Value	TGF-β1 Expression (%, Spearman r)	*p*-Value
Postoperative IKDC score	−0.46	0.009	−0.42	0.015
Postoperative Lysholm score	−0.41	0.018	−0.38	0.026

**Table 4:** Postoperative clinical outcomes according to MMP-13 and TGF-β1 expression levels.

Outcome	Low MMP-13	High MMP-13	*p*-Value	Low TGF-β1	High TGF-β1	*p*-Value
Postoperative IKDC score, median (IQR)	86 (80–90)	78 (72–84)	0.012	85 (79–89)	79 (73–83)	0.021
Postoperative Lysholm score, median (IQR)	92 (88–95)	86 (80–90)	0.018	91 (87–94)	86 (81–90)	0.029
Positive pivot-shift, n (%)	1 (6.7)	6 (40.0)	0.028	1 (6.7)	5 (33.3)	0.047
Graft failure, n (%)	0 (0)	3 (20.0)	0.070	0 (0)	2 (13.3)	0.140

## Data Availability

The datasets generated and analyzed during the current study are available from the corresponding author upon reasonable request.

## Supplementary Materials

The following supporting information can be downloaded at: https://www.mdpi.com/article/10.3390/medicina62030457/s1, Figure S1: Box–whisker plot showing the distribution of QuPath-derived MMP-13 H-scores in acute and chronic ACL remnant tissues; Figure S2: Box–whisker plot illustrating the distribution of QuPath-derived TGF-β1 H-scores in acute and chronic ACL remnant tissues; Figure S3: Arthroscopic appearance of anterior cruciate ligament (ACL) remnant tissue in acute and chronic injuries; Table S1: Network centrality analysis of the shared-protein PPI network.
