# Supplementary material for: Chronic ACL Injury Drives a Fibrotic and Matrix-Degradative Shift: A Multi-Level Analysis of MMP-13 and TGF-β1
Source: Medicina (Kaunas). 2026 Feb 27;62(3):457. doi: 10.3390/medicina62030457 (PMC13028566; doi:10.3390/medicina62030457)
Supplement: Supplementary file 1 [file medicina-62-00457-s001.zip › medicina-4051207-supplementary.pdf]

Supplementary Figures of MMP-13 and TGF-β Expression H-Scores

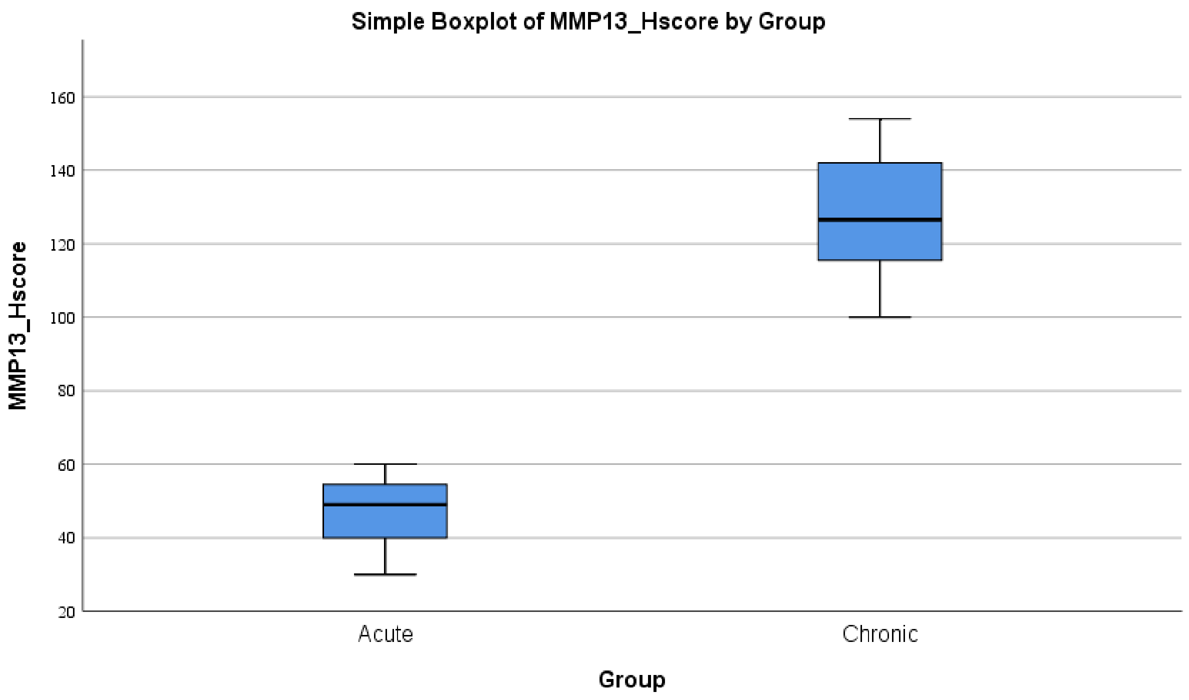

Figure S1. Box–whisker plot showing the distribution of QuPath-derived MMP-13 H-scores in acute and chronic ACL remnant tissues. The box represents the interquartile range, the central line indicates the median, and whiskers denote minimum and maximum values. Chronic ACL remnants exhibit markedly higher MMP-13 expression compared with acute injuries.

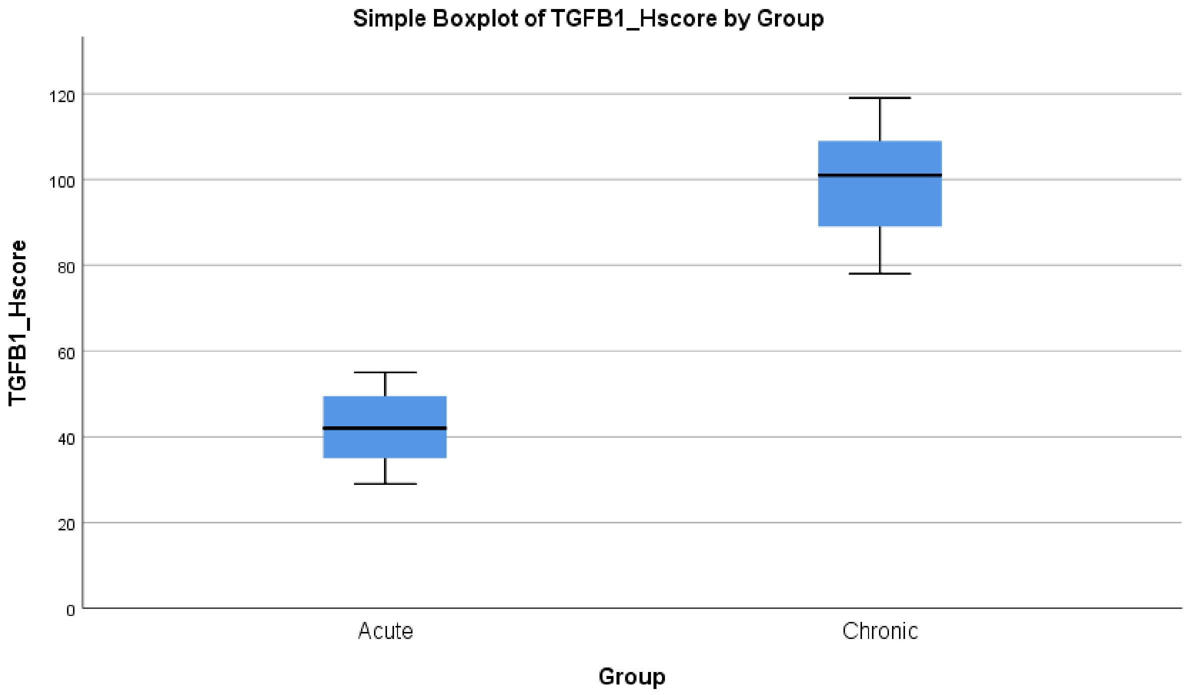

Figure S2. Box–whisker plot illustrating the distribution of QuPath-derived TGF-β1 H-scores in acute and chronic ACL remnant tissues. Boxes represent the interquartile range with the median indicated by the central line, while whiskers denote minimum and maximum values. Chronic ACL remnants demonstrate significantly higher TGF-β1 expression compared with acute injuries.

Acute ACL injury (<3 months) demonstrating a relatively preserved remnant structure with continuous, well-defined ligament fibers and a soft, voluminous appearance. The remnant tissue shows a smooth surface and maintained fiber organization, consistent with an early reparative microenvironment and limited degenerative change (Figure S3A). Chronic ACL injury (>6 months) showing marked morphological degeneration characterized by bulky, irregular, and disorganized remnant tissue. The ligament fibers appear thickened, poorly defined, and fibrotic, with loss of normal fibrillar architecture,

reflecting chronic extracellular matrix remodeling and fibrotic transformation (Figure S3B).

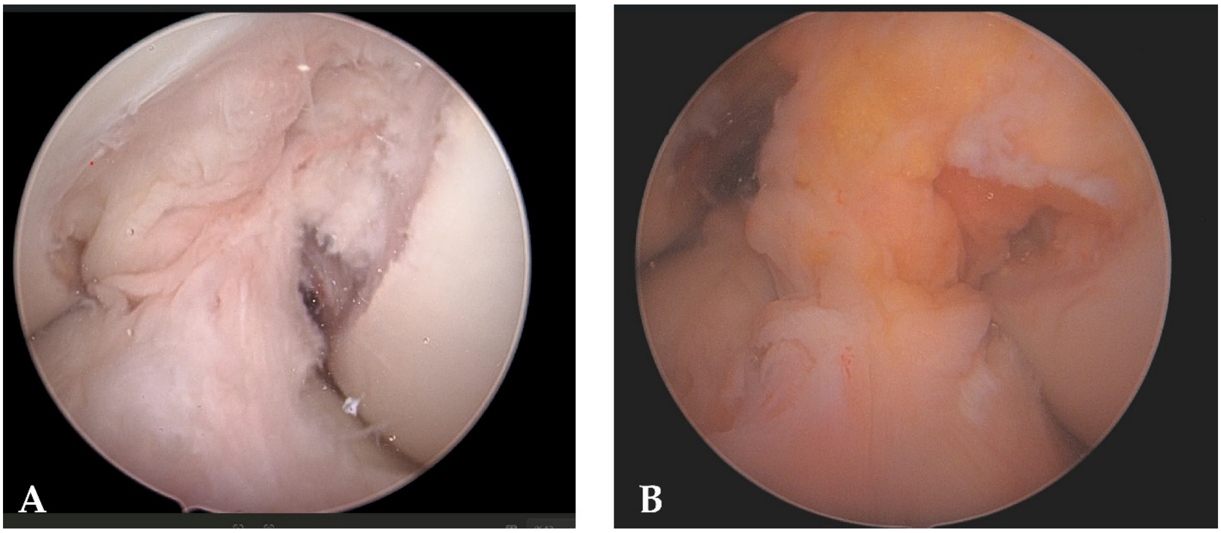

Figure S3. Arthroscopic appearance of anterior cruciate ligament (ACL) remnant tissue in acute and chronic injuries. A: Acute ACL injury (<3 months), Chronic ACL injury (>6 months).

Supplementary Table S1. Network centrality analysis of the shared-protein PPI network.

Protein Name	Degree	Betweenness
COL2A1	21	0.0080
COL3A1	27	0.0230
COL11A1	19	0.0036
LOXL2	9	0.0001
COL1A2	29	0.0300
BMP1	21	0.0139
TGF-β1	32	0.0678
MMP-12	21	0.0102
POSTN	24	0.0115
LUM	23	0.0119
MMP-13	36	0.1126
MMP-7	23	0.0336
MMP-1	26	0.0190
MMP-10	10	0.0019
COL10A1	20	0.0070
COL18A1	19	0.0063
ELN	27	0.0395
CTSK	20	0.0069
PRSS1	6	0.0000
PLG	16	0.0124
FMOD	22	0.0099
MMP20	10	0.0020
TGFβR1	9	0.0000
ADAMTS5	17	0.0051
LTBP3	6	0.0008
MMP-3	31	0.0376
ADAMTS4	20	0.0070
COMP	20	0.0033
ADAMTS3	18	0.0079
MMP-19	9	0.0012
MMP-16	7	0.0006
TGF-β2	17	0.0133
OPTC	2	0.0000
ACAN	26	0.0202
CCN2	24	0.0121

IBSP	14	0.0049
MMP14	22	0.0165
IHH	7	0.0001
LTBP4	4	0.0000
